# Transient cortical visual impairment after video-assisted thoracic surgery: a case report

**DOI:** 10.1186/s12886-015-0157-1

**Published:** 2015-11-17

**Authors:** Hee Kyung Yang, Jeong-Min Hwang

**Affiliations:** Department of Ophthalmology, Seoul National University College of Medicine, Seoul National University Bundang Hospital, 166, Gumiro, Bundang-gu, Seongnam, Gyeonggi-do 463-707 Korea

**Keywords:** Transient, Cortical visual impairment, Video-assisted thoracic surgery

## Abstract

**Background:**

Visual loss associated with thoracic surgery has been reported mostly after coronary angiography or bypass surgery. The position of video-assisted thoracic surgery (VATS) is usually lateral, thus not compressive to the globe. Visual loss after VATS has not been reported. Herein we report a patient without any cardiovascular risk factors who experienced transient cortical blindness after an uneventful VATS.

**Case presentation:**

A 40-year-old man noticed a visual loss at the recovery room after VATS. He showed normal pupillary reflex, normal optic disc appearance, and homonymous hemianopia respecting the vertical meridian, thus was typical for cortical visual impairment.

**Conclusions:**

Transient cortical visual impairment could be encountered after an uneventful VATS in a patient without any cardiovascular risk factors.

## Background

Visual loss associated with thoracic surgery has been reported mostly after coronary angiography or bypass surgery [1]. Hypotension, anemia, emboli, and small vessel inflammation may increase the risk of visual loss [2]. In addition, surgeries with prone position may cause sustained compression of the globe leading to central retinal artery or vein occlusion [3]. The position of video-assisted thoracic surgery (VATS) is usually lateral, thus not compressive to the globe. Visual loss after VATS has not been reported. Herein we report a patient without any cardiovascular risk factors who experienced transient cortical blindness after an uneventful VATS.

## Case presentation

A 40-year-old man was referred from the Department of Thoracic Surgery with visual loss noticed at the recovery room after VATS. He underwent VATS for posterior segmentectomy of the right upper lobe and left lower lobe for lung masses under general anesthesia. VATS was uneventful and took approximately 2 hours without any significant blood pressure fluctuation or blood loss. The histologic examination confirmed malignant lymphoma and reactive hyperplasia in surrounding lymph nodes. His blood pressure, hemoglobin, glucose and cholesterol levels were all within normal ranges.

On ophthalmologic evaluation immediately after surgery, his visual acuities were hand motion in both eyes (OU). Pupils were reactive without any relative afferent pupillary defect. Eye motility was full OU. Slit lamp examination of eyelid and anterior chamber and fundus examination were normal. Brain magnetic resonance imaging (MRI) including diffusion weighted imaging (DWI) and angiography showed no abnormal findings. He was alert without confusion and did not show any cognitive abnormalities.

**Fig. 1:**
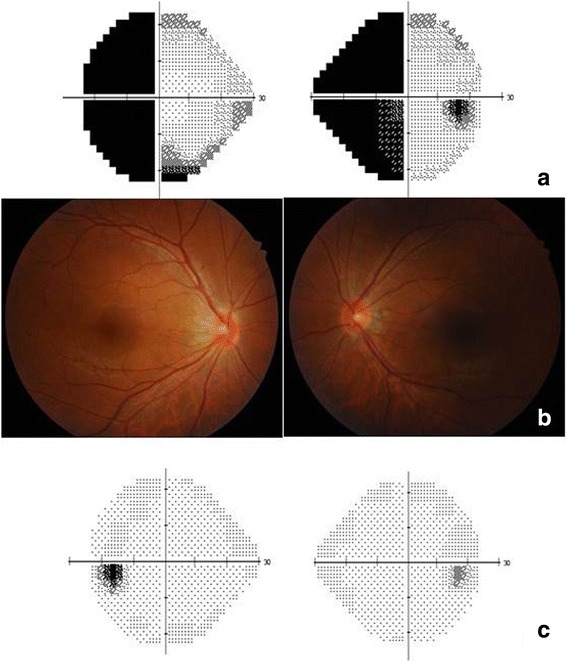
Ophthalmologic findings. **a** Humphrey visual field testing showed left homonymous hemianopia one day after video-assisted thoracic surgery (VATS). **b** Fundus photographs disclosed no abnormal findings one day after VATS. **c** Humphrey visual field testing returned to normal two day after VATS.

One day after VATS, visual acuities improved to 8/200 OU. Hardy-Rand-Rittler color test showed total achromatopsia OU. Intraocular pressures were 14 mmHg OD and 18 mmHg OS. Humphrey perimetry showed left homonymous hemianopia (Fig. 1a). Pupillary examination, slit lamp and fundus examination were normal (Fig. 1b). Two days after VATS, visual acuities were 20/20 OU. Hardy-Rand-Rittler color test and Humphrey perimetry returned to normal (Fig. 1c).

## Discussion

The mechanism of visual loss after thoracic surgery is, in most cases, ischemia especially of the optic nerve or occipital lobe. Ischemia of the optic nerve may present as anterior or posterior optic neuropathy which could be differentiated with the presence of optic disc edema and hemorrhages. Ischemia of the occipital lobe may present as cortical visual impairment which could be differentiated from optic neuropathy by the presence of normal pupillary light reflex [4]. Our patient showed normal pupillary light reflex, normal optic disc appearance, and homonymous hemianopia respecting the vertical meridian, thus was typical for cortical visual impairment.

Cortical visual impairment after thoracic surgery may be caused by emboli, systemic hypotension, or anemia [5]. Our patient showed a rapid recovery within 2 days and conditions that may cause reversible cortical blindness include a transient ischemic attack (TIA) and posterior reversible encephalopathy syndrome (PRES). TIA is a transient neurologic deficit with a rapid recovery lasting within a few minutes or less than 24 hours that is attributed to focal ischemia of the brain or retina [6]. DWI is sensitive in the detection of acute events of small and early infarcts, and a positive DWI lesion is a reliable predictor or recurrent stroke [7]. About 40 % of patients with TIA have been reported to have a positive DWI lesions, however, our patient was negative on DWI [7]. TIA is usually caused by a small emboli and is often associated with hypertension, heart disease, hyperlipidemia and diabetes mellitus [6]. As our patient did not have any of these risk factors, a small emboli to the posterior cerebral artery or transient ischemia of the occipital lobe by vascular compromise could be the plausible cause of cortical visual impairment. PRES is a syndrome characterized by headache, confusion, seizures and visual loss, which may occur due to malignant hypertension, eclampsia or surgery [8]. MRI findings are typical with hyperintense signals in T2-weighted imaging, fluid attenuated inversion recovery (FLAIR) or DWI due to subcortical and cortical edema in the occipital and parietal regions related to posterior cerebral artery supply [8]. However, the clinical and radiologic findings of our case do not suggest PRES.

The loss of vision after thoracic surgery is transient in most of the cases, lasting from seconds to hours, but could also be persistent. Visser et al. [9] reported loss of consciousness accompanied by persistent cortical blindness after an injection of thoracic epidural test dose of bupivacaine. They did not provide the exact visual acuity or presence of pupillary light reflex, but the patient could virtually “see nothing”. Brain MRI showed increased signal intensity with vasogenic cerebral edema in the occipital lobe, suspecting infarction in the areas supplied by the posterior cerebral artery.

## Conclusion

Transient cortical visual impairment could be encountered after an uneventful VATS in a patient without any cardiovascular risk factors.

## Consent

Written informed consent was obtained from the patient for publication of this case report and any accompanying images. A copy of the written consent is available for review by the Editor of this journal. Approval by the IRB was exempted as this was a single case report.
